# Life history and fitness costs in the lambda‐cyhalothrin resistant clone of English grain aphid, *Sitobion avenae* (Fabricius)

**DOI:** 10.1002/ps.8924

**Published:** 2025-05-29

**Authors:** Md Munir Mostafiz, Ali Güncan, Maximilian Schughart, Stephen Byrne, Louise McNamara

**Affiliations:** ^1^ Teagasc, Crop Science Department Carlow Ireland; ^2^ Department of Plant Protection, Faculty of Agriculture Ordu University Ordu Türkiye

**Keywords:** age‐stage, two‐sex life table, grain aphid, insecticide resistance, life‐history trait

## Abstract

**BACKGROUND:**

The English grain aphid, *Sitobion avenae* (Fabricius) (Hemiptera: Aphididae), is a highly detrimental cereal aphid in Western Europe, infesting all cereal crops, including barley, wheat and oats. Pyrethroid pesticides are extensively utilized for the management of *S*. *avenae* worldwide. The extended use of pyrethroid pesticides to manage *S. avenae* has led to the emergence of resistance to lambda‐cyhalothrin. However, a detailed assessment of life‐history traits linked to lambda‐cyhalothrin resistance in *S. avenae* has not yet been conducted. The objectives of this study were to utilize age‐stage, two‐sex life table theory to examine the life‐history traits of a widespread clonal lineage of *S. avenae* that exhibits partial resistance to lambda‐cyhalothrin.

**RESULTS:**

There was a significant decrease in the relative fitness (0.508) of lambda‐cyhalothrin‐resistant clones (SA3) in comparison to the susceptible clonal lineage (SA27) of *S. avenae*. The biological parameter data indicated that the SA3 clonal lineage exhibited significantly prolonged developmental stages (excluding N4) and reduced longevity, fecundity, adult pre‐reproductive period, and reproductive days compared to the SA27 clone. By contrast, population growth parameters, including the net reproduction rate (*R*_0_), intrinsic rate of increase (*r*) and finite rate of increase (*λ*), were markedly lower in the SA3 clone than in the SA27 clone of *S. avenae*.

**CONCLUSIONS:**

These data provide comprehensive insights into the life‐history characteristics and population dynamics of the lambda‐cyhalothrin‐resistant clone of *S. avenae*, which may be fundamental for development of decision support tools and resistance management measures against this pest. © 2025 The Author(s). *Pest Management Science* published by John Wiley & Sons Ltd on behalf of Society of Chemical Industry.

## Graphical abstract

The purpose of this study is to investigate the life‐history traits and fitness costs of English grain aphid clones that are resistant to lambda‐cyhalothrin (SA3) and susceptible to it (SA27). Overall, the results showed that there are differences in the stages of development, survival, reproduction and population growth.

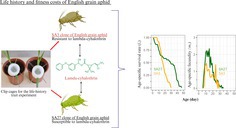

## 1 INTRODUCTION

*Sitobion avenae* (Fabricius) (Hemiptera: Aphididae), commonly known as the English grain aphid, is a predominant cereal aphid species in Europe and other regions of the world, feeding on all cereal crops, including barley, wheat, oats, rice and grasses.^1^ This aphid species causes significant reductions in grain production by directly extracting plant sap and facilitating the spread of plant viruses.^2^, ^3^ In particular, *S*. *avenae* is a key vector of plant viruses, including the barley yellow dwarf virus (BYDV), which reduces yield losses by >80% in cases of high infection.^4^ Chemical control is recognized as one of the most effective methods for managing grain aphids in agricultural production. Pyrethroid insecticides are employed all over the world for the management of grain aphids.^5^, ^6^ The extensive and regular use of pyrethroid insecticides in agriculture significantly promotes the development of several forms of pesticide resistance in grain aphids.^7^, ^8^, ^9^

Lambda‐cyhalothrin is a pyrethroid insecticide recognized for its ability to interfere with the neurological system of insects by affecting voltage‐gated sodium channels, as well as calcium and chloride channels.^10^ Consequently, it is extensively utilized in the management of insect pests, including *S. avenae*,^5^, ^7^ *Myzus persicae* (Sulzer),^11^ *Rhopalosiphum padi* (L.),^12^ *Helicoverpa armigera* (Hiibner)^13^ and *Spodoptera exigua* (Hübner).^14^

In Europe, a clonal lineage of *S. avenae* that exhibited resistance to lambda‐cyhalothrin was designated as the heterozygous SA3 clone.^5^ This clonal lineage was first discovered in 2011 in England and Scotland, and was later identified in Ireland in 2013.^5^, ^15^ *Kdr* (knock‐down resistance) is a target‐site mutation that is involved in the resistance process. It is one of the most common ways that insects may resist pyrethroids.^16^ Furthermore, the *kdr* mutation has been shown to lead to lambda‐cyhalothrin resistance in many aphid species, including *R*. *padi*, *M*. *persicae* and *Aphis glycines* (Matsumura).^12^, ^17^, ^18^, ^19^ Mutations in key target genes may lead to various impacts on phenotypic and biological characteristics.^20^ It is also recognized that insecticide resistance reduces fitness by frequently altering important life‐history characteristics that are essential to population dynamics, such as reproduction, development and survival.^20^, ^21^, ^22^ According to Mokbel *et al*.,^22^ fitness costs characterize an organism's ability to survive and transmit its biological characteristics to future generations. The development of pesticide resistance incurs a substantial energy cost for the insect that can reduce fitness within the insecticide‐resistant population.^23^ Numerous studies have documented fitness costs associated with various forms of insecticide resistance in a range of insect species, *R. padi*,^12^ *Aphis craccivora* Koch,^22^ *Aphis gossypii* Glover,^24^ *Halotydeus destructor* (Tucker),^25^ *Bemisia tabaci* (Gennadius),^21^ *Cydia pomonella* (L.)^26^ and *S. avenae*.^27^ Understanding the influence of fitness costs on life‐history traits is essential for predicting the potential implications that resistance might have for disease outbreaks.

So far, only a few studies have been conducted on the reproductive fitness and sexual potential of the heterozygous‐resistant clone (SA3) of *S. avenae*.^15^, ^27^, ^28^ However, in the majority of the studies, the impact of the heterozygous‐resistant clone (SA3) of *S. avenae* in pesticide‐free environments on biological traits (developmental durations, preadult survival rate, reproductive days, longevity and fecundity) and population parameters, which typically influence relative fitness, were not examined. Rapid resistance evolution is particularly dependent on relative fitness. The relative fitness differences between the susceptible clone (SA27) and resistant clone (SA3) of *S. avenae* are not well‐understood. To evaluate the relative fitness cost, this study aimed to investigate the impact of partial lambda‐cyhalothrin resistance on the life‐history traits of *S. avenae*. The age‐stage, two‐sex life table method was employed to precisely assess the demographic traits of a susceptible (SA27) and a heterozygous‐resistant (SA3) clonal lineage of *S. avenae*. The age‐stage life table approach enables a detailed description of the development, survival, reproduction and life expectancy of populations. In addition to providing a comprehensive description of the stage differentiation (also known as metamorphosis), the methodology accounts for preadult survival by including all individuals.

## 2 MATERIALS AND METHODS

### 2.1 Insect collection and colony‐rearing

The aphid colonies were maintained in nylon mesh insect rearing cages (32.5 × 32.5 × 32.5 cm) within a growth chamber set at 24 °C and 55% relative humidity (RH), with a 16:8 h, light:dark photoperiod. The insecticide‐resistant clonal lineage SA3 was derived from a single aphid collected in 2017 from winter wheat in County Carlow, Ireland, while the insecticide‐susceptible clonal lineage SA27 originated from a single aphid collected in 2021 from winter barley in the same location. Winter barley (*Hordeum vulgare* L. cv. LG Casting) served as the host plant for both colony upkeep and the experiment. Barley seeds were planted in 7.5‐cm plastic pots containing John Innes Compost/No2 and then placed in a growth chamber until reaching the age required for colony maintenance.

### 2.2 Aphid genotyping and *kdr* testing

The aphids were genotyped by the James Hutton Institute, Invergowrie, Scotland, following a published protocol and five microsatellite loci (Sm10, SM12, Sm17, Sa∑4 and S16b).^29^, ^30^ Additionally, five adult aphids from both SA3 and SA27 colonies were tested for the *kdr* mutation L1014F before the experiments, using an established TaqMan PCR protocol from Rothamsted Research, UK.^5^, ^29^ For this, DNA was extracted from individual aphids using a sucrose buffer extraction method, as described by Louis.^31^

### 2.3 Lambda‐cyhalothrin bioassays

Insecticide bioassays were conducted to test the two clonal lineages (SA3 and SA27) for their respective survival rates after an insecticide exposure. For this, newly emerged adult apterous aphids (within 24 h) for both clonal lineages were exposed to lambda‐cyhalothrin in a single‐dose bioassay in scintillation glass vials at the recommended field rate for pyrethroids (5 g ha^−1^ active ingredient of lambda‐cyhalothrin) or in glass vials without any insecticide as a control. To evaluate metabolic resistance, additional bioassays were conducted using glass vials coated with both piperonyl butoxide (PBO) and lambda‐cyhalothrin (0.58 μL/100 cm^2^ active ingredient PBO). A control group was exposed to PBO‐coated vials without insecticide. To coat the vials, the respective amount of lambda‐cyhalothrin and PBO were diluted in acetone, and 500 μL were pipetted into a scintillation glass vial (38.56 cm^2^ surface; Wheaton, Millville, NJ, USA). Each vial was placed on a roller in a fume hood at room temperature for 2 h, to allow acetone to evaporate. For each treatment, 12 aphids were introduced into a vial, and their movement was observed at different time intervals: 5, 10, 15, 20, 30 and 45 min, as well as at 1, 2, 4, 6 and 24 h post‐exposure. The experiment was conducted with four replicate vials (each with 12 aphids) per treatment for each aphid clone.

The Cox proportional hazards model was utilized to analyze survival data and assess the impact of treatment groups on aphid mortality over time. Survival time was defined as the time until mortality occurred (event), with censored data representing aphids that survived until the end of the study period. Hazard ratios (HRs) along with 95% confidence intervals (CIs) were computed to determine the relative risk of mortality for each clone within the treatments. To verify the adequacy of the Cox model, the proportional hazards assumption was tested using Schoenfeld residuals, with a nonsignificant global test result indicating that the assumption was satisfied. Survival curves were generated using the Kaplan–Meier method. All analyses were performed using the _SURVIVAL_ ^32^ and the graph was created with survminer ^33^ package in R.^34^

### 2.4 Evaluating the life‐history trait of *S. avenae* on winter barley

**Figure 1:**
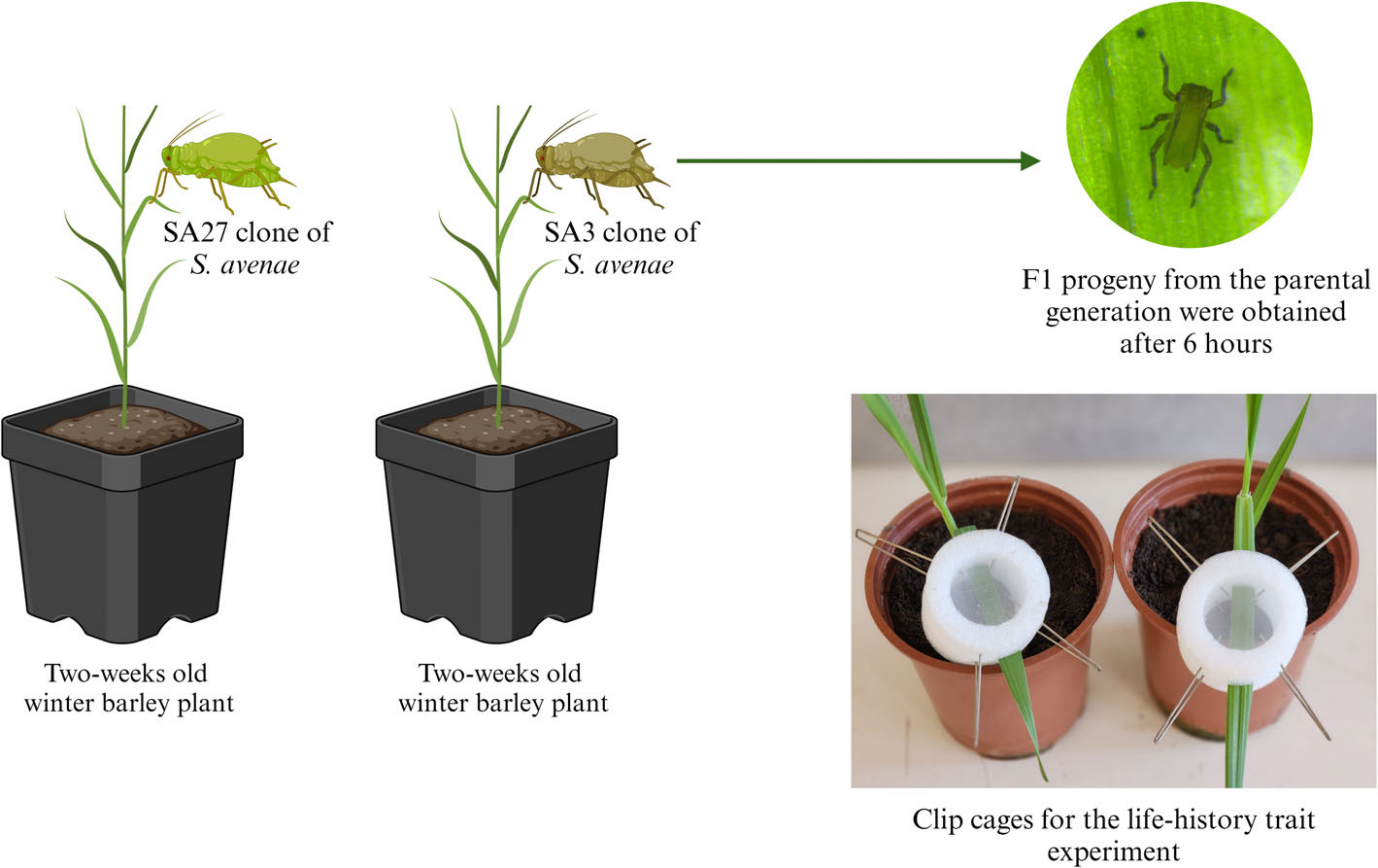
Experimental design for the life‐history traits of susceptible clone (SA27) and resistant clone (SA3) of *Sitobion avenae* reared on winter barley in a clip cage.

The demographic characteristics of the susceptible clone (SA27) and the resistant clone (SA3) of *S. avenae* were revealed by utilizing the life table experiments. The experiment began with planting barley seedlings, one in each pot, as described in Section 2.1. These plants were used for the life‐history assessment. To produce an experimental cohort of 1^st^‐instar nymphs, 36 wingless female adults of each clone were each placed individually in a clip cage (NHBS, Bonn, Germany) after the barley plants reached the two‐leaf stage. The clip‐cage methodology was used as described by Hass *et al*.^35^ to restrain very small insects on leaves to investigate their biological characteristics. While it has been reported that clip‐cages can influence aphid behavior in some species,^1^, ^36^ a recent study by Kou *et al*.^37^ demonstrated that clip‐cages did not adversely affect the reproduction or development of *S. avenae*, the species used in this study. To minimize potential stress on the aphids, we used clip‐cages with adequate ventilation and monitored the aphids daily to confirm normal feeding and development behaviors. Within a period of 6 h, the plants were examined and a fine paintbrush was used to remove all the aphids from each pot, except for one freshly emerged 1^st^‐instar nymph. One clip cage was used for each plant, and 36 1^st^‐instar nymphs were chosen for each of the SA27 and SA3 clones (Fig. 1). In this study, each individual aphid was treated as a separate replicate, with a total of 36 replications assessed for each clonal lineage of *S. avenae*. Data on development time, lifespan, fecundity and mortality were collected daily until all aphids died. During the reproductive stage, the number of offspring produced by each female was counted and removed daily. All plants, equipped with clip cages containing aphids from both clonal lineages, were placed in a growth chamber maintained at 24 °C with 55% RH and a 16:8 h, light:dark photoperiod.

### 2.5 Life table data analysis

The age‐stage life table approach was applied to analyze the life‐history data obtained from the SA27 and SA3 clones of *S*. *avenae* according to established methods.^38^, ^39^, ^40^ While *S*. *avenae* mostly reproduces parthenogenetically, the age‐stage life table approach accounts for varying growth rates across individuals and stage difference. This approach considers not only the stage of differentiation but also suggests a solid relationship between the net reproductive rate (*R*_0_) and the fecundity (*F*).^40^ This approach has been utilized to evaluate the age‐stage specific survival rate (*s*_*xj*_), age‐specific survival rate (*l*_*x*_), age‐stage life expectancy (*e*_*xj*_) and reproductive value (*v*_*xj*_), alongside biological parameters including developmental duration, adult longevity, and fecundity for each individual aphid utilizing the TWOSEX‐MSChart program.^41^, ^42^ Population traits such as the intrinsic rate of increase (*r*), the finite rate of increase (*λ*), the net reproductive rate (*R*_0_) and the mean generation time (*T*), were also computed using the TWOSEX‐MSChart program. The variances and standard errors of all life table parameters were calculated using the bootstrap approach with 100 000 bootstrap samples.^43^ The paired bootstrap test was used to assess the differences in all life table parameters between the SA27 and SA3 clonal lineages at a 5% significance level.^44^ The graphs were created with the ggplot2 package^45^ using R.^34^

## 3 RESULTS

### 3.1 Survival rates of SA27 and SA3 clonal lineages of *S. avenae* after exposure to lambda‐cyhalothrin and lambda‐cyhalothrin+PBO

**Figure 2:**
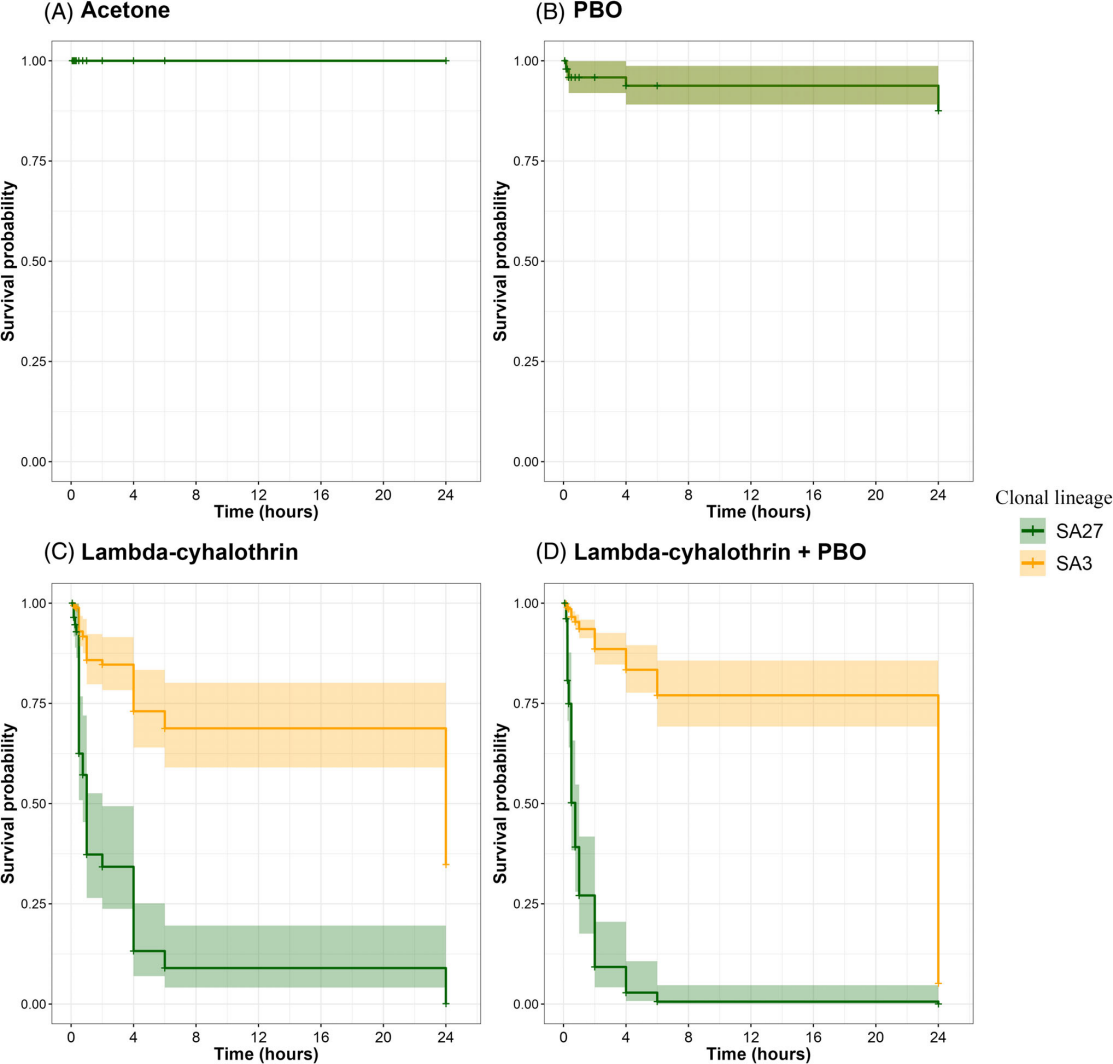
Kaplan–Meier survival curves for the susceptible clone SA27 (dark green) and the lambda‐cyhalothrin‐resistant clone SA3 (orange) of *Sitobion avenae* treated with (A) Acetone, (B) PBO, (C) Lambda‐cyhalothrin, and (D) Lambda‐cyhalothrin + PBO, with 95% confidence intervals (shaded areas).

After molecular testing, the presence of *kdr* mutation in the SA3 clonal lineage, and the absence of *kdr* in the SA27 clonal lineage was confirmed in all aphids tested from the respective colonies. Both SA3 and SA27 clonal lineages of *S. avenae* were exposed to four treatment conditions: (A) control (acetone), (B) PBO alone, (C) 100% field rate of lambda‐cyhalothrin, and (D) 100% field rate of lambda‐cyhalothrin + PBO. Survival analysis using the Cox proportional hazards model revealed distinct responses of aphid clones SA3 and SA27 to different treatments (Fig. 2). No mortality was observed in either aphid clone (SA3 and SA27) when exposed to the acetone control treatment, indicating that acetone itself had no adverse effect on aphid survival [Fig. 2(A)]. Likewise, aphids of both clonal lineages showed high nonsignificant survival rates >95% after a 6 h exposure to PBO alone, yet after 24 h of exposure to PBO alone, the mean survival rate slightly decreased to 83.33% in the SA3 clone and 87.50% in the SA27 clone [Fig. 2(B)]. However, when the clones where exposed to a 100% field rate of lambda‐cyhalothrin, the survival rates after 6 h exposure in the insecticide resistant clonal lineage SA3 (72.92%) were significantly higher in comparison to the SA27 clonal lineage (6.25%). After a 24 h 100% field rate exposure, the SA3 clone exhibited a substantially lower mortality risk compared to SA27 (Hazard Ratio, HR = 0.155, 95% CI: 0.101–0.238, *P* < 0.001), indicating that SA3 is more tolerant to lambda‐cyhalothrin. While no SA27 aphids survived, 33.33% of SA3 aphids remained alive [Fig. 2(C)]. This was also the case when the aphids were exposed to a combination of lambda‐cyhalothrin + PBO [Fig. 2(D)]. After a 6 h exposure to both chemicals, none of the SA27 aphids survived, while the SA3 clone showed high survival rates, with 77.08% of aphids remaining alive. However, after a 24 h exposure to lambda‐cyhalothrin + PBO, the survival rate of the SA3 clone decreased to 4.17%. Despite this, under the combined treatment of lambda‐cyhalothrin and PBO, the SA3 clone exhibited a significantly reduced mortality risk compared to SA27 (HR = 0.051, 95% CI: 0.043–0.061, *P* < 0.001) [Fig. 2(D)].

### 3.2 Developmental duration and longevity of SA27 and SA3 clonal lineages of *S*. *avenae*

Table 1 provides biological data on the developmental duration, adult longevity, and fecundity of SA27 and SA3 clones of *S. avenae*. The mean developmental durations for the 1^st^‐, 2^nd^‐ and 3^rd^‐instar nymphs of SA3 clones were notably longer compared to those of SA27 clones. However, no significant differences in developmental time were observed for the 4^th^‐instar nymphs (*P* = 0.68916) (Table 1). The SA3 clones had a substantially longer pre‐adult stage (N1 to N4) (8.88 days) than the SA27 clones (7.69 days) (*P* = 0.00165). The SA27 clone demonstrated slightly higher mortality across the nymphal stages, as reflected in the declining number of individuals completing each stage (Table 1). The pre‐adult survival rates were 80.56% for SA27 and 88.89% for SA3, with no significant difference between the two clonal lineages (*P* = 0.32621). However, compared to SA3, SA27 had a considerably greater mean adult longevity (*P* < 0.00001) and a significantly higher total longevity (*P* < 0.00001). The total lifetime of the clonal lineages of *S*. *avenae* was found to be 25.42 days for SA27 and 17.72 days for SA3 (Table 1).

**Table 1:** Duration (days) of different developmental stages (Mean ± SE) of susceptible clone (SA27) and lambda‐cyhalothrin‐resistant clone (SA3) of *Sitobion avenae* reared on winter barley

Stages	Clones of *Sitobion avenae*				
	SA27		SA3		*P*‐value
	*n*	Mean ± SE	*n*	Mean ± SE	
1^st^‐instar nymph (N1)	35	2.06 ± 0.10b	36	2.33 ± 0.09a	0.04131
2^nd^‐instar nymph (N2)	32	1.75 ± 0.14b	35	2.31 ± 0.09a	0.00071
3^rd^‐instar nymph (N3)	30	1.50 ± 0.10b	33	1.94 ± 0.14a	0.01234
4^th^‐instar nymph (N4)	29	2.52 ± 0.13a	32	2.44 ± 0.15a	0.68916
Pre‐adult (N1 to N4)*	29	7.69 ± 0.23b	32	8.88 ± 0.30a	0.00165
Pre‐adult survival rate (*s*_*a*_) (%)	36	80.56 ± 6.5a	36	88.89 ± 5.2a	0.32621
Adult longevity	29	22.66 ± 2.23a	32	9.94 ± 0.94b	<0.00001
Total longevity (all individuals)	36	25.42 ± 2.49a	36	17.72 ± 1.00b	<0.00001

Standard errors were calculated with the bootstrap method with 100 000 bootstrap samples. The paired bootstrap test was used to compare the differences. The means within a row followed by a different letter indicate a significant difference between the clones.

* The pre‐adult stage in the age‐stage life table refers to the developmental period from the 1^st^‐instar (N1) to the 4^th^‐instar (N4) nymphal stage, before the individual reaches adulthood.

### 3.3 Reproduction and demographic parameters of SA27 and SA3 clonal lineages of *S. avenae*

Table 2 presents the reproduction and key demographic parameters of the SA27 and SA3 clones of *S*. *avenae*. The results showed that SA3 had a significantly longer total pre‐reproductive period (TPRP) (9.63 days) compared to SA27 (8.79 days) (*P* = 0.0299). Nonetheless, both SA27 and SA3 clones exhibited no significant effects (*P* = 0.87534) throughout the adult pre‐reproductive period (APRP). The highest number of reproductive days was observed for SA27 (10.90 days), significantly above that of SA3 (6.30 days) (*P* = 0.00012). Additionally, SA27 had a considerably greater fecundity rate (22.21 nymphs/female) than SA3 (10.22 nymphs/female) (*P* < 0.00001). Furthermore, the main demographic parameters, namely *R*_0_, *r* and *λ*, each exhibited an increase in the SA27 clone of *S*. *avenae* relative to the SA3 clone. Significant variations were identified between the two clones across all three demographic factors (Table 2). However, there was no significant difference in the mean generation time (*T*) between the clones (*P* = 0.56373). The relative fitness (*R*_f_), derived from the *R*_0_ values in the life table, was 0.508 times lower for the SA3 clone than for the SA27 clone, which suggests that fitness costs may be associated with lambda‐cyhalothrin resistance in the SA3 clone.

**Table 2:** Reproduction and life table parameters (Mean ± SE) of the susceptible clone (SA27) and lambda‐cyhalothrin‐resistant clone (SA3) of *Sitobion avenae* reared on winter barley

Parameters*	Clones of *Sitobion avenae*		
	SA27	SA3	*P* value
	Mean ± SE	Mean ± SE	
*R*_0_ (offspring/individual)	17.89 ± 2.39a	9.08 ± 1.12b	0.00078
*r* (day^−1^)	0.2131 ± 0.0113a	0.1668 ± 0.0092b	0.00197
*λ* (day^−1^)	1.2375 ± 0.0139a	1.1815 ± 0.0108b	0.00197
*T* (days)	13.53 ± 0.34a	13.23 ± 0.43a	0.56373
*F* (nymphs/female)	22.21 ± 2.35a	10.22 ± 1.11b	<0.00001
RP_*d*_ (days)	10.90 ± 1.03a	6.30 ± 0.58b	0.00012
APRP (days)	1.10 ± 0.18a	1.07 ± 0.14a	0.87534
TPRP (days)	8.79 ± 0.27b	9.63 ± 0.28a	0.02999
*R*_f_ ^†^	—	0.508	—

Standard errors were calculated with the bootstrap method with 100,000 bootstrap samples. Comparisons of differences were conducted using the paired bootstrap test (*P* < 0.05). The means within a row denoted by a distinct letter signify a considerable variation among the clones.

* *R*_0_ = net reproductive rate; *r* = intrinsic rate of increase; *λ* = finite rate of increase; *T* = mean generation time; *F* = fecundity; RP_*d*_ = reproductive days; APRP = adult pre‐reproductive period; TPRP = total pre‐reproductive period.

^†^ *R*_f_ = *R*_0_ of the resistant clone (SA3)/*R*_0_ of the susceptible clone (SA27), *R*_f_ > 1 suggests that the fecundity of resistant clone is enhanced, whereas *R*_f_ < 1 suggests that the resistant clone has a fitness cost.^46^

### 3.4 Survival rate and fecundity of SA27 and SA3 clonal lineages of *S. avenae*

**Figure 3:**
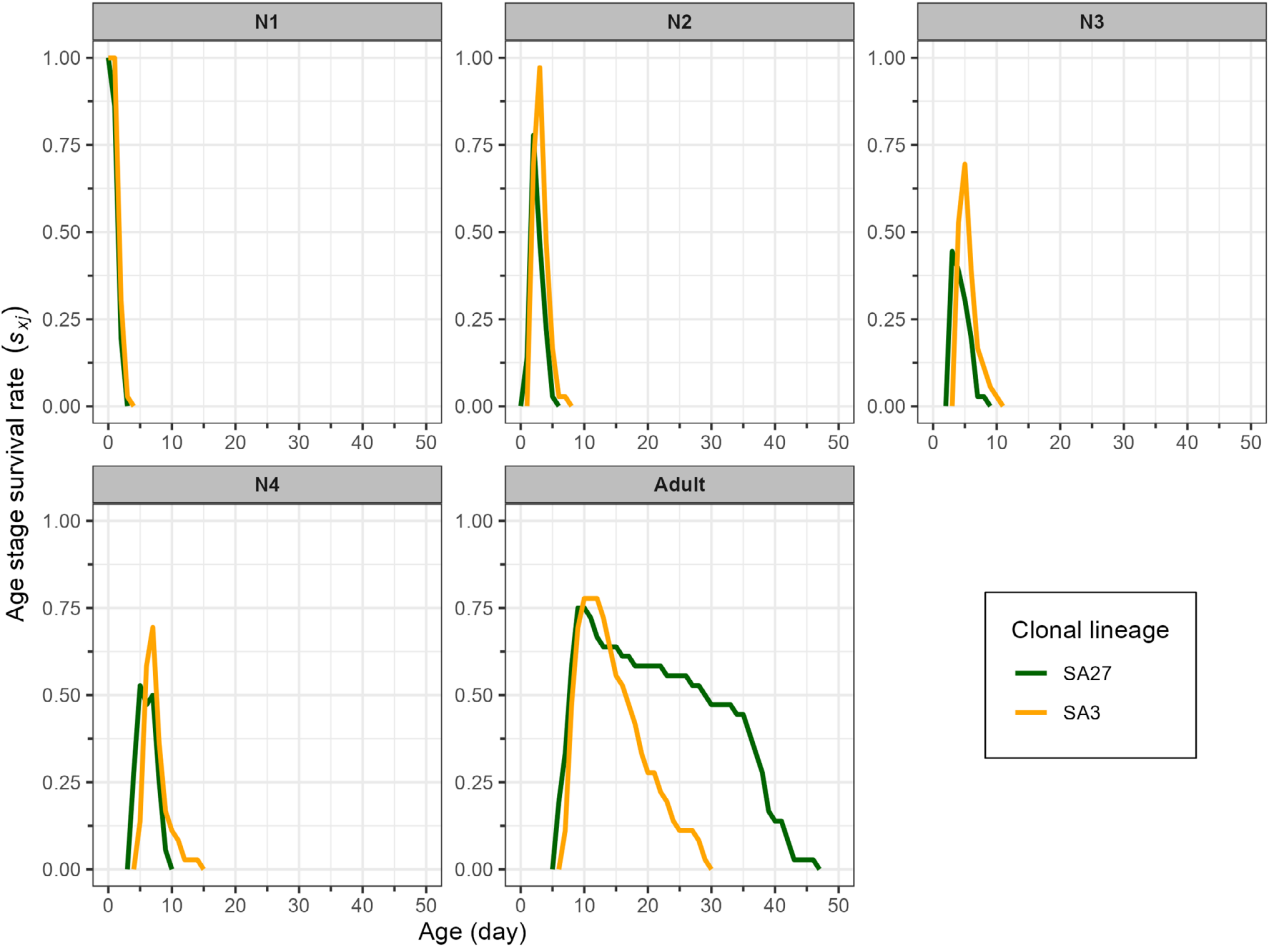
Age‐stage specific survival rate (*s*_*xj*_) of susceptible (SA27) and resistant (SA3) clones of *Sitobion avenae* on winter barley plants. N1–N4 denote the 1^st^‐ to 4^th^‐instars of *S*. *avenae*.

Fig. 3 illustrates the age‐stage survival rate (*s*_*xj*_), exhibiting significant overlaps across various stages owing to the developmental stage variability among individuals of the SA27 and SA3 clonal lineages. The female adults emerged at 6 and 7 days, respectively, for the SA27 and SA3 clones (Fig. 3). An increased lifespan of female adults of SA27 was also seen in comparison to the SA3 clones (Fig. 3).

**Figure 4:**
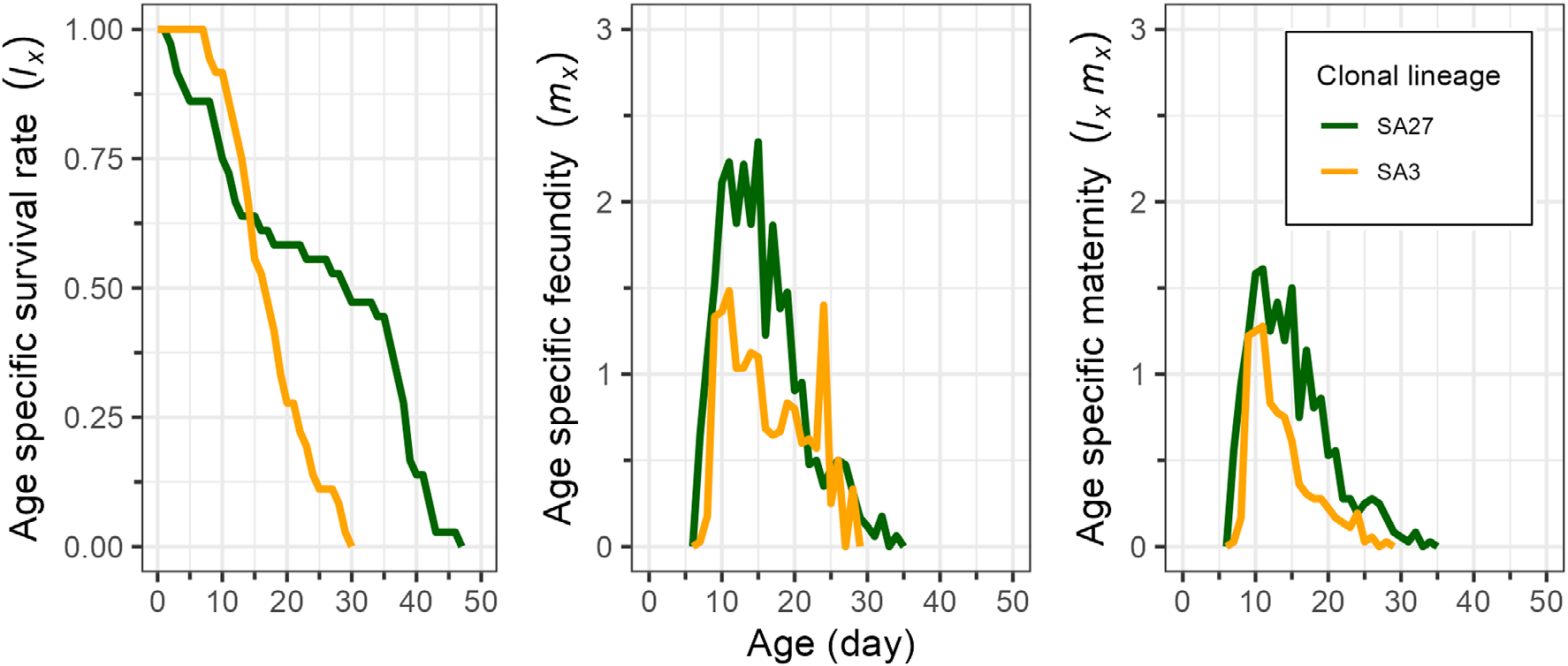
Age‐specific survival rate (*l*_*x*_), age‐specific fecundity (*m*_*x*_) and age‐specific maternity (*l*_*x*_ *m*_*x*_) of susceptible (SA27) and resistant (SA3) clones of *Sitobion avenae* on winter barley plants.

Fig. 4 illustrates the age‐specific survival rates (*l*_*x*_), age‐specific fecundities of the entire population (*m*_*x*_) and age‐specific maternity (*l*_*x*_ *m*_*x*_) for SA27 and SA3. Compared to the SA3 clone, the *l*_*x*_ curve of the SA27 clone showed a considerable decline beginning at age 5 days, whereas the SA3 clone's curve showed a decrease beginning at age 9 days (Fig. 4). In comparison to the SA3 clone, the *l*_*x*_ value decreased at an earlier stage for the SA27 clone. As shown in Fig. 4, the maximum *m*_*x*_ peaks for SA27 clone occurred at age 15 days (with 2.35 offspring), whereas those for SA3 clone occurred at age 11 days (with 1.48 offspring). According to both *l*_*x*_ and *m*_*x*_, SA27 exhibited a peak *l*_*x*_ *m*_*x*_ value of 1.5 offspring at 15 days, whereas SA3 demonstrated a value of 1.28 offspring at 11 days (Fig. 4).

### 3.5 Life expectancy and reproductive value of SA27 and SA3 clonal lineages of *S. avenae*

**Figure 5:**
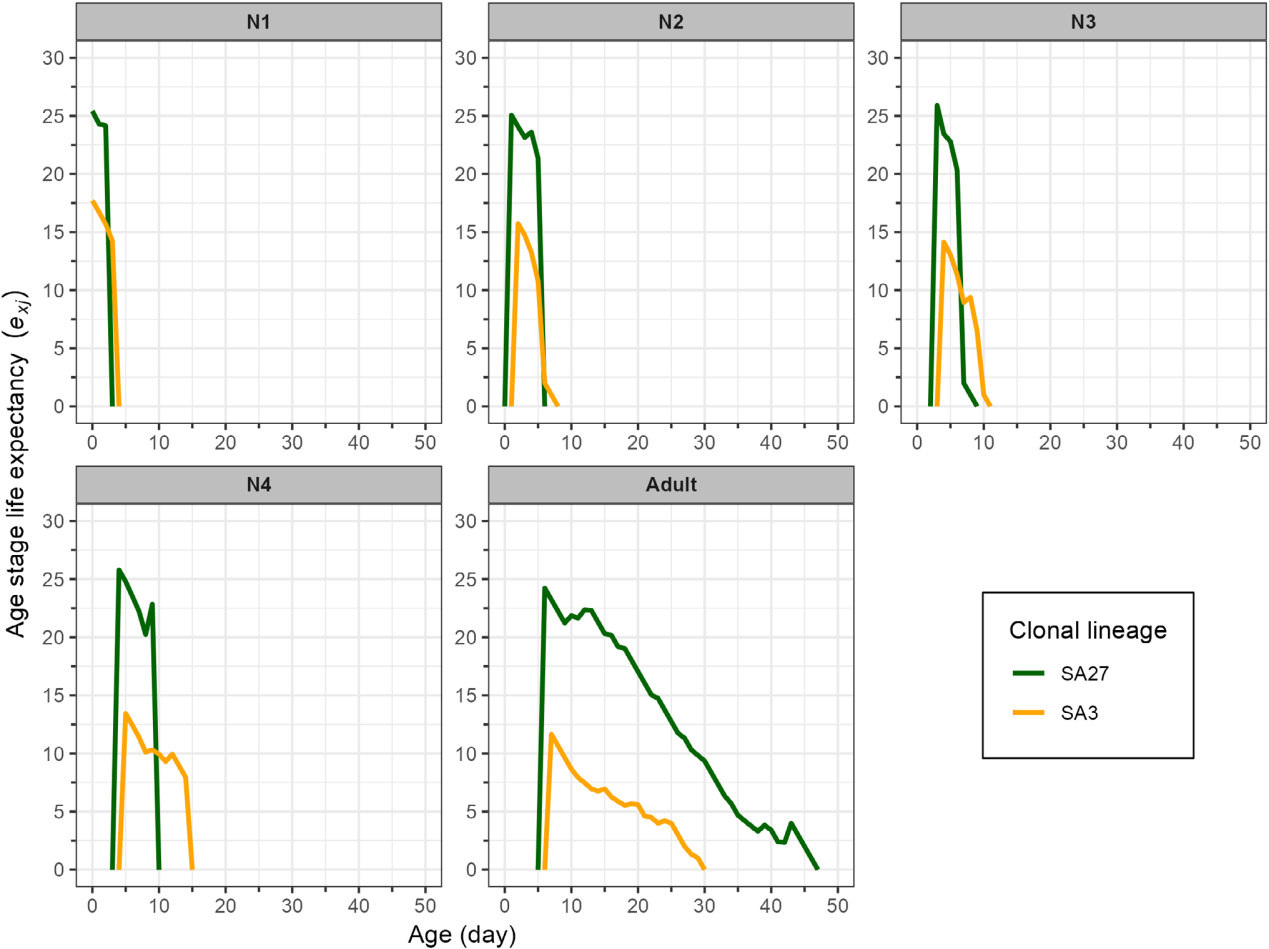
Age‐stage specific life expectancy (*e*_*xj*_) of susceptible (SA27) and resistant (SA3) clones of *Sitobion avenae* on winter barley plants. N1–N4 denote the 1^st^‐ to 4^th^‐instars of *S. avenae*.

The age‐stage‐specific life expectancy (*e*_*xj*_) curves clearly indicated that the life expectancy of the SA27 clone was greater than that of SA3. For a newly born nymph, the life expectancy was 25.42 days for SA27 and 17.72 days for SA3 (Fig. 5).

**Figure 6:**
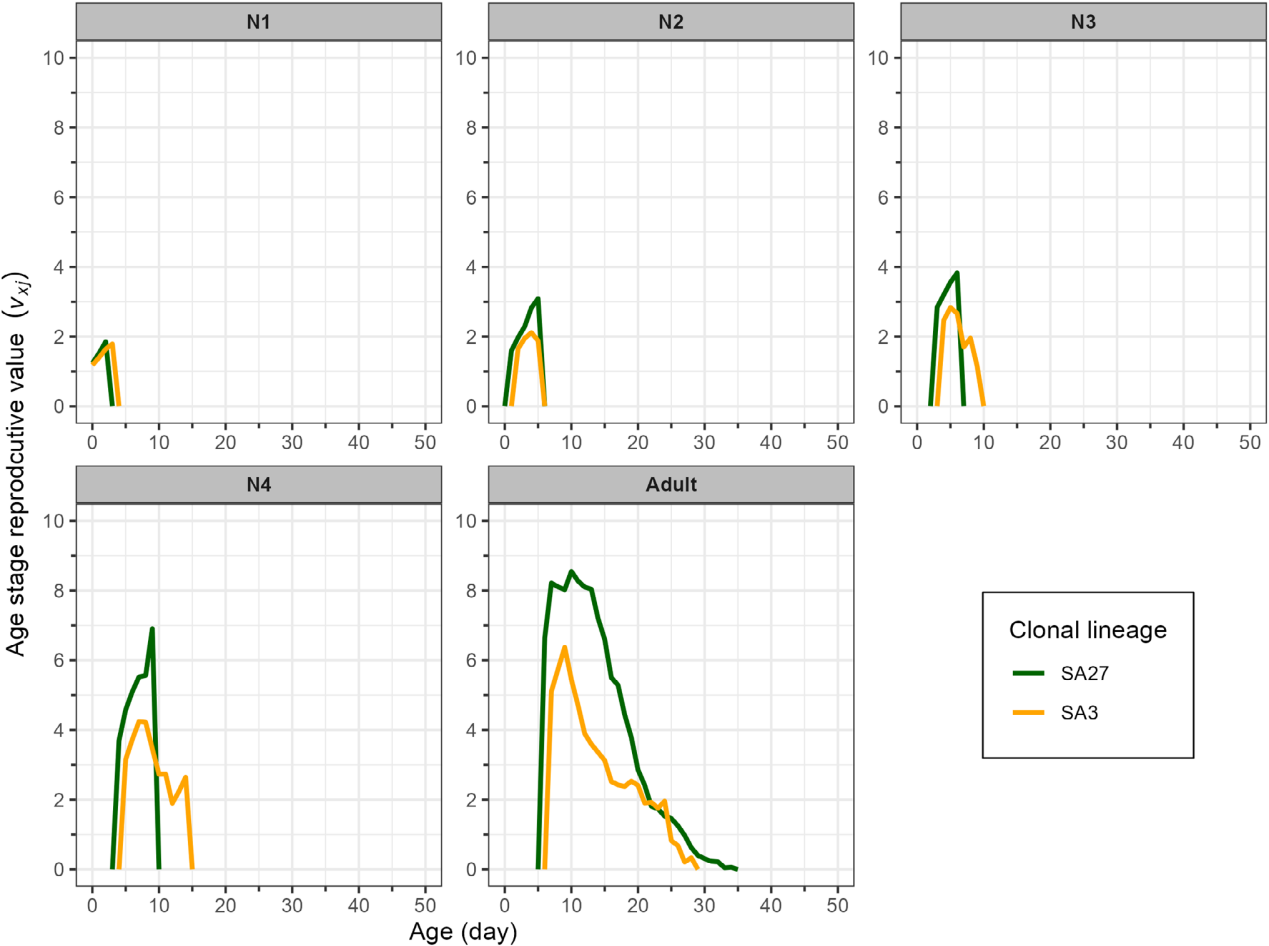
Age‐stage specific reproductive value (*v*_*xj*_) of susceptible (SA27) and resistant (SA3) clones of *Sitobion avenae* on winter barley plants. N1–N4 denotes the 1^st^‐ to 4^th^‐instars of *Sitobion avenae*.

The reproductive value (*v*_*xj*_) represents the projected contribution of an individual of age *x* and stage *j* to the future population. The peak reproductive values for SA27 and SA3 clones were observed at ages 10 days (*v*_10, Adult_ = 8.55) and 9 days (*v*_9, Adult_ = 6.37), respectively (Fig. 6).

## 4 DISCUSSION

Chemical pesticides are widely used for aphid control. However, the establishment of pesticide resistance has diminished the efficacy of insecticides.^47^, ^48^ Numerous species of aphids have displayed resistance to multiple pesticides,^7^, ^8^, ^9^ including *S. avenae*, which has been reported to exhibit resistance to lambda‐cyhalothrin.^5^, ^15^ No prior study has examined the fitness costs associated with lambda‐cyhalothrin resistance in *S. avenae* using the age‐stage, two‐sex life table theory. It is appropriate to evaluate the related fitness costs in *S. avenae* to understand the threat that resistant clones pose in disease outbreaks and inform appropriate resistance management strategies.

In line with a previous study,^5^ our findings in the insecticide exposure bioassays suggest that the primary mode of resistance to lambda‐cyhalothrin in the *S. avenae* clone SA3 originates from the *kdr*‐mutation. This is shown by similarly high survival rates of the SA3 clone after a 6 h exposure to either a 100% field rate of lambda‐cyhalothrin or to lambda‐cyhalothrin + PBO in combination. Nonetheless, the survival rates of SA3 aphids after a 24 h exposure to lambda‐cyhalothrin + PBO are lower than in SA3 aphids that were exposed only to lambda‐cyhalothrin. However, this is not unexpected, as PBO is a well‐established inhibitor of cytochrome P450 monooxygenases, esterases and glutathione S‐transferases (GSTs), which are critical enzymes in metabolic resistance mechanisms, but whose inhibition can cause stress and death after a long‐time exposure.^49^ In many aphid species, enhanced detoxification enzyme activity previously has been linked to resistance against pyrethroids, including lambda‐cyhalothrin.^50^, ^51^ Moreover, the upregulation of detoxification enzymes is likely to impose an energetic cost on resistant populations, potentially affecting their reproductive fitness and competitive ability in the absence of insecticidal pressure.^52^ However, as the SA3 aphids in this study were collected in 2017 and have not been exposed to any insecticides for >8 years (or 300+ generations), we propose that the upregulation of detoxification enzymes does not play a significant role in resistance to lambda‐cyhalothrin in this clonal lineage. Nonetheless, further studies quantifying specific enzyme activities (e.g. esterase titers or GST levels) could clarify their relative roles in resistance evolution in *S. avenae*.

There are typically fitness trade‐offs associated with the evolution of pesticide resistance in insects. This phenomenon indicates that individuals resistant to insecticides may experience costs in the absence of insecticides.^19^, ^23^ The costs of fitness significantly influence the development of insecticide resistance, as resistant individuals often exhibit reduced fitness compared to susceptible individuals in pesticide‐free environments.^19^, ^27^, ^53^ Biological traits, including prolonged development length and decreased fecundity and reproductive rate, usually impact relative fitness.^54^ The relative fitness of *S. avenae* (0.508) significantly decreased in the lambda‐cyhalothrin‐resistant clone (SA3) in comparison to the susceptible clone (SA27), indicating that the fitness cost may be associated with resistance to lambda‐cyhalothrin. For the majority of the time, these costs are the consequence of physiological trade‐offs, such as greater metabolic demands or disturbances in important biological activities as a result of mutations that confer resistance.^23^ It is possible, for instance, for target‐site changes and increased detoxification impose energy costs, which in turn reduces the reproductive success, lifespan or competitiveness of the insect in the absence of pesticide treatment.^23^

Additionally, our results showed that the development of 1^st^‐, 2^nd^‐ and 3^rd^‐instar was delayed in the SA3 clone compared to the SA27 clone. It was shown that the resistant clone had a much longer pre‐adult duration compared to the susceptible clone. These results align with prior research indicating that individuals of *A. gossypii* resistant to imidacloprid had significantly longer developmental and pre‐adult stages compared to susceptible individuals.^24^ Thiamethoxam‐resistant strains of *A*. *gossypii* and *R*. *padi* exhibited an extended developmental duration.^9^, ^55^ Additionally, in *C. pomonella* and *Grapholita molesta* (Busck), the resistant clones notably had a prolonged larval duration in response to lambda‐cyhalothrin.^26^, ^56^ In another study, Gul *et al*.^57^ revealed that the developmental durations of *Bradysia odoriphaga* (Yang and Zhang) were markedly extended in clothianidin‐resistant strains relative to susceptible populations. Based on the data presented here, an extended developmental period appears to be one of the most significant fitness costs associated with pesticide resistance in target insect pests. Consequently, it is possible that clones of *S. avenae* with resistance to lambda‐cyhalothrin may not proliferate rapidly in the field in the absence of pesticide selection pressure, especially given the fitness costs observed in this study, which may reduce their competitiveness compared to non‐resistant individuals. However, in the presence of lambda‐cyhalothrin, resistant clones gain a selective advantage, allowing them to proliferate rapidly as non‐resistant individuals are eliminated.^58^

The growth potential of an insect population may be more accurately evaluated via the examination of the intrinsic rate of increase in life table analysis. It is a key measure of an organism's potential for population growth under ideal conditions, representing the rate at which a population would grow if there were no limiting factors. The present findings indicated that the intrinsic rate of increase was markedly reduced in SA3 clones relative to SA27 clones, suggesting that the SA27 population has a higher potential for rapid growth. This information is important for understanding the dynamics of pest infestations and has direct implications for pest management. Specifically, knowing the intrinsic rate of increase allows for the prediction of population growth trends, enabling timely and targeted control measures. A higher intrinsic rate suggests the need for earlier intervention to prevent outbreaks, whereas a lower rate may indicate a slower population buildup, allowing for extended monitoring intervals. For example, biological control agents (e.g. predators) could be deployed preemptively against SA27‐dominated populations, as their slower‐acting nature demands timing adjustments to match rapid aphid growth. Alternatively, combining biological controls with resistant crop varieties may better suppress high‐*r* clones. This knowledge ultimately enhances the efficiency and effectiveness of aphid management strategies.

The findings of this study provide insights for developing sustainable pest management strategies. For example, life‐history traits can feed into degree‐day models,^59^ which accumulate thermal units to predict the development and emergence of pest populations. These models can be refined using the life‐history parameters identified in this study. For *S. avenae*, industry‐standard models (e.g. Bayer's T‐Sum Calculator)^60^ use a threshold temperature (*T*_0_) of 3 °C and calculate accumulated degree days (DD), where DD = (*T*–*T*_0_). A threshold of 170 DD is then used to indicate the emergence of second wingless aphid generation, indicating pyrethroid treatment. In our study, the SA27 and SA3 clones reared at 24 °C (*T* = 24 °C) developed to reproductive adulthood in 8.79 and 9.63 days, respectively. When calculated using the same *T*_0_ (3 °C), this equates to 161.5 DD for SA27 and 186.2 DD for SA3; with the current threshold of 170 DD falling within this range. With these clone specific DD values, agronomists can use local temperature data to predict when specific aphid populations will reach peak reproductive activity. For example, by accumulating DD sums from biofix points (e.g. first spring aphid detection), interventions such as insecticide applications or predator releases can be timed to target vulnerable life stages before populations exceed economic thresholds.

In conclusion, the *S. avenae* SA3 clone exhibits a fitness cost that may be associated with its lambda‐cyhalothrin resistance, characterized by an extended developmental period, reduced lifespan, lower fecundity and a decreased intrinsic rate of increase in comparison to the susceptible clone (SA27).

## Data Availability

The data that support the findings of this study are available from the corresponding author upon reasonable request.
